# Genome-wide association mapping and genomic prediction for kernel color traits in intermediate wheatgrass (*Thinopyrum intermedium*)

**DOI:** 10.1186/s12870-022-03616-7

**Published:** 2022-04-28

**Authors:** Prabin Bajgain, Catherine Li, James A. Anderson

**Affiliations:** 1 Department of Agronomy and Plant Genetics, University of Minnesota, St. Paul, MN 55108 USA; 2 Department of Crop Sciences, University of Illinois, Urbana-Champaign, IL 61801 USA

**Keywords:** Intermediate wheatgrass, Perennial food crop, Kernel color, Genetic mapping, Haplotype, Genomic selection

## Abstract

**Background:**

Intermediate wheatgrass (IWG) is a novel perennial grain crop currently undergoing domestication. It offers important ecosystem benefits while producing grain suitable for human consumption. Several aspects of plant biology and genetic control are yet to be studied in this new crop. To understand trait behavior and genetic characterization of kernel color in IWG breeding germplasm from the University of Minnesota was evaluated for the CIELAB components (L*, a*, b*) and visual differences. Trait values were used in a genome-wide association scan to reveal genomic regions controlling IWG’s kernel color. The usability of genomic prediction in predicting kernel color traits was also evaluated using a four-fold cross validation method.

**Results:**

A wide phenotypic variation was observed for all four kernel color traits with pairwise trait correlations ranging from − 0.85 to 0.27. Medium to high estimates of broad sense trait heritabilities were observed and ranged from 0.41 to 0.78. A genome-wide association scan with single SNP markers detected 20 significant marker-trait associations in 9 chromosomes and 23 associations in 10 chromosomes using multi-allelic haplotype blocks. Four of the 20 significant SNP markers and six of the 23 significant haplotype blocks were common between two or more traits. Evaluation of genomic prediction of kernel color traits revealed the visual score to have highest mean predictive ability (*r*^*2*^ = 0.53); *r*^*2*^ for the CIELAB traits ranged from 0.29–0.33. A search for candidate genes led to detection of seven IWG genes in strong alignment with MYB36 transcription factors from other cereal crops of the Triticeae tribe. Three of these seven IWG genes had moderate similarities with *R-A1*, *R-B1*, and *R-D1*, the three genes that control grain color in wheat.

**Conclusions:**

We characterized the distribution of kernel color in IWG for the first time, which revealed a broad phenotypic diversity in an elite breeding germplasm. Identification of genetic loci controlling the trait and a proof-of-concept that genomic selection might be useful in selecting genotypes of interest could help accelerate the breeding of this novel crop towards specific end-use.

**Supplementary Information:**

The online version contains supplementary material available at 10.1186/s12870-022-03616-7.

## Background

Intermediate wheatgrass [IWG, *Thinopyrum intermedium* (Host) Barkworth & D.R. Dewey subsp. *intermedium*, 2n = 6x = 42] was introduced to North America as a forage crop in the 1930s [1]. The crop is a cool-season perennial grass species and is being domesticated as a novel food crop by multiple institutions including the University of Minnesota (UMN). Initiated in 2011, the UMN’s IWG breeding and domestication program has completed four breeding cycles and released a food-grade IWG cultivar named ‘MN-Clearwater’ in 2019 [2, 3]. Besides the use of IWG grain as food, the crop also can provide substantial services towards ecosystem preservation such as improved carbon sequestration, reduced nutrient runoff and leaching to groundwater, and reduced soil erosion [4, 5].

As a novel grain crop undergoing active domestication efforts, many genetic advancements are yet to be made in IWG. These advancements towards both the continued improvement of agronomic traits and the exploration of end-use traits will make the crop more desirable to farmers, processors, and consumers. Examples of these important traits include grain yield, seed size, plant height, lodging resistance, and disease resistance, and domestication traits such as shatter resistance and free grain threshing [2, 6–8]. Variation for seed color also has been observed in UMN populations, ranging from light sandy tones to deep purples. In wheat, the genes influencing seed color are known to produce antioxidants that influence flavor, nutritional qualities [9], and seed dormancy [10]. This has also been observed in corn [11], rice [12], and barley [13]. Thus, similar relations are expected in IWG, and trait characterization and genetic mapping of loci influencing seed color traits will help breeders and geneticists make informed decisions regarding these traits. For example, in a study by Ma et al. [14], seed color in wheat was found to be significantly correlated with antioxidant activity, phenolic, carotenoid and flavonoid contents, and grain weight. Purple wheat had the highest quantities of these phytochemicals and antioxidant activity, followed by red wheat and white wheat. However, the correlation between the aforementioned traits and grain weight was found to be negative, meaning darker grains had lower grain weights. Another specialty grain, purple corn, is currently on the market as a superfood, enticing consumers with a unique taste and higher antioxidant content than blueberries [15, 16]. The outcomes of this study could therefore impact the selection of ideotypes with desired kernel color in the University of Minnesota IWG breeding program and have strong implications in food-related use of IWG grain.

In a diverse breeding population such as the one discussed in this study, the methodology predominantly used to uncover the genetic architecture controlling a trait is association mapping, and is typically implemented in the form of a genome-wide association study (GWAS). For a comprehensive and successful GWAS, it is preferable to have abundant markers distributed across the genome that represent the overall genomic diversity. Such discovery can be used to select individuals with preferred genetic makeup and also to discover candidate genes in genomic regions controlling the trait [17]. The availability of a high-quality, annotated genome sequence is therefore a major determinant in discovery of abundant, quality genome-wide markers (often SNPs, single nucleotide polymorphisms) and success of GWAS. Because of their abundance in a genome, SNPs can be used to infer the genome-wide allelic diversity and LD structure, which are instrumental in conducting robust and accurate GWAS along with the identification of causative loci and candidate genes [18]. In addition to SNPs, haplotype blocks are also recommended for GWAS [19, 20]. Haplotypes are combinations of many SNP alleles that tend to be co-inherited. While SNPs are the smallest genetic units used in GWAS for mapping, larger units such as haplotype blocks are also increasingly being used in gene mapping studies as they provide multi-allelic information whereas SNPs only provide bi-allelic information [21]. Haplotype blocks therefore enable a better investigation of complex mechanisms of causal genes, gene sets or pathways, rather than a single locus [22].

Genomic selection is an in vivo marker-based selection approach where information across the whole genome is used to evaluate a population of interest. Statistical models, often known as genomic prediction models, compute the influence of genome-wide markers in traits of interest and have been shown to be a robust method to improve genetic gain and breeding efficiency in crops [23, 24] including IWG [8, 25]. Genomic selection can also be an effective tool to select superior genotypes when selecting for quantitative traits and traits controlled by several loci of small to medium effects [26]. In our IWG breeding program, we have demonstrated the usefulness of genomic prediction models to predict yield and yield component traits [7], domestication-related traits [27], and disease resistance traits [6]. Thus, if effective in predicting IWG kernel color, genomic prediction models can save time and resources by predicting the trait in large breeding populations and greatly reduce the need for field trials and phenotyping.

We therefore conducted this study with the primary goal of uncovering the genetic architecture of kernel color in IWG. Towards this goal, we evaluated our elite breeding germplasm for four kernel color traits and investigate the relationships among these traits, and their distributions and heritabilities. We used single SNP markers and multi-allelic haplotype blocks to identify genomic regions controlling kernel color in IWG. We also evaluated the feasibility of genomic selection in selecting IWG genotypes for kernel color traits by using a genomic prediction model to predict the trait value.

## Results

### Trait distribution and properties

**Fig. 1:**
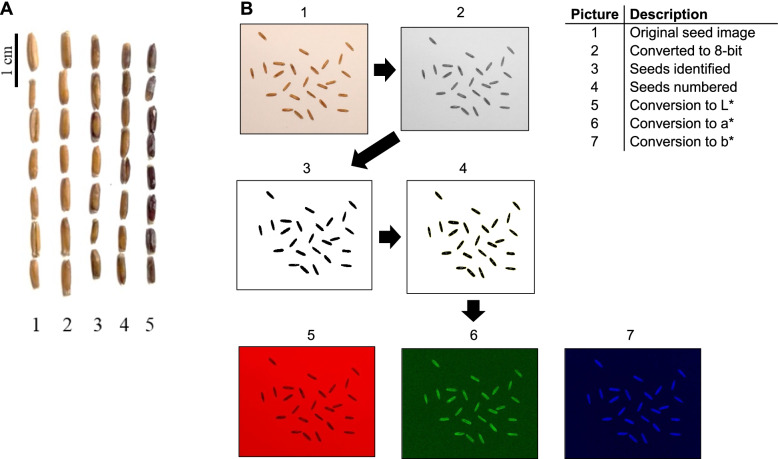
**A** Range of kernel colors observed in the University of Minnesota’s IWG breeding program. Seed are from mother plants of the fourth breeding cycle population grown in St. Paul, 2020. Numbers below each column of seed show a visual scale (V) of manual color assignment. **B** The process of image analysis and extraction of L*, a*, and b* values using ImageJ. The L*, a*, and b* values were measured for each seed in each sample

**Fig. 2:**
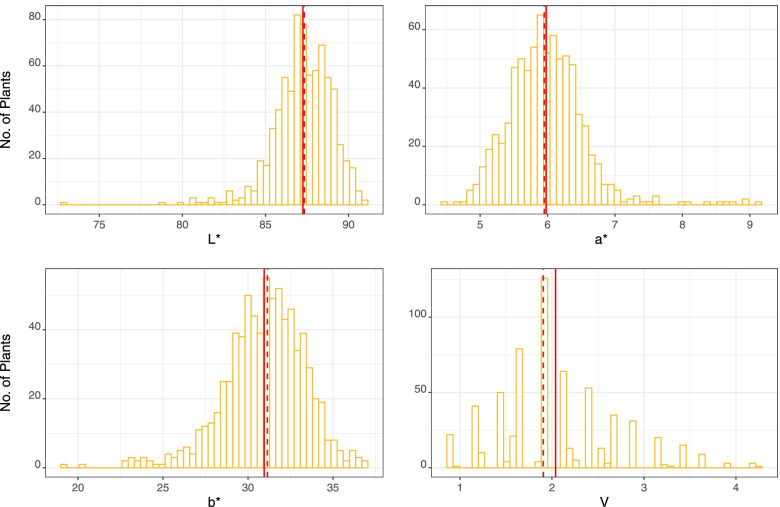
Kernel color trait values, i.e. best linear unbiased estimates (BLUEs), in the UMN_C4 intermediate wheatgrass breeding population. Four traits were measured: three according to the CIELAB (L*, a*, b*) system and one visually (V). The vertical dashed red lines indicate trait median and solid red lines indicate trait mean values

Phenotypic data on IWG kernel color was obtained from ImageJ (CIELAB values) and visual rating of the grain (Fig. 1). A broad variability for the four kernel color traits was observed in in the UMN_C4 IWG population (Fig. 2). The mean BLUE values for L*, a*, b*, and V* were 87.3, 6.0, 31.0, and 2.0, respectively. Broad sense trait heritability estimates (H) were medium to high with the highest for visual score: H = 0.78. For the CIELAB traits L*, a*, and b*, H estimates were 0.45, 0.53, and 0.41, respectively. Narrow-sense heritability estimates were overall high for all four traits with averages of 0.84, 0.81, 0.77, and 0.70 for V, b*, a*, and L*, respectively. Variance components used in estimation of H as well as narrow-sense heritabilities are provided in Additional File 1.

The strongest pairwise trait correlation was observed between L* and a* (*r* = − 0.85, Table 1). The CIELAB trait b* had relatively weak yet positive and significant correlation with L* (*r* = 0.27) and negative but significant correlation with V (*r* = − 0.12). Visual scores (V) was positively and significantly correlated with L* but had negative relationship with both a* and b*.

**Table 1:** Pairwise Pearson correlation coefficients among the four kernel color traits in the UMN_C4 intermediate wheatgrass population. Three traits (L*, a*, b*) were measured according to the CIELAB system and one was assessed visually (V). The symbol * next to the correlation coefficient values indicates significance at *α* = 0.05

Traits	L*	a*	b*
a*	−0.85*		
b*	0.27*	0.02	
V	0.22*	−0.04	−0.12*

### Genome-wide association analysis

**Fig. 3:**
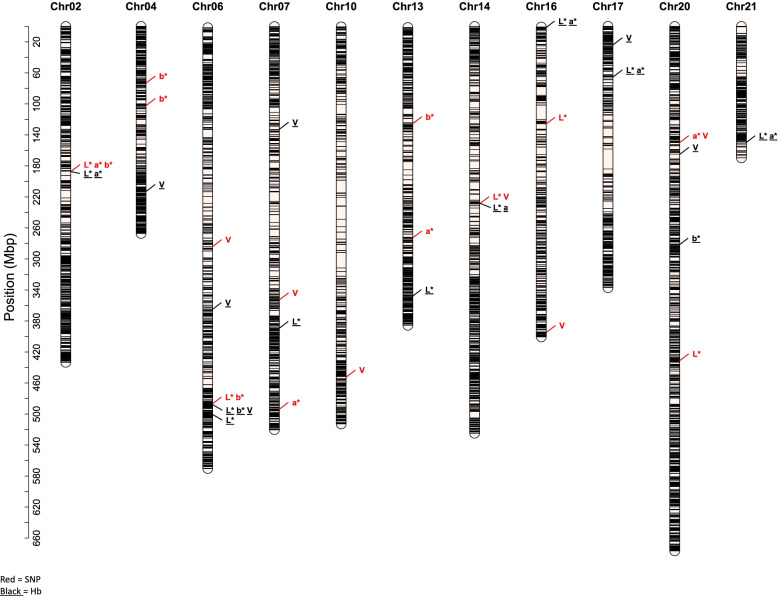
Distribution of SNP markers and haplotype blocks in significant association with the CIELAB kernel color traits L*, a*, b*, and visual score (V). Red colored bars and letters show positions of SNP markers and black underlined bars and letters show positions of haplotype blocks. The scale on the left shows physical marker positions in mega base pairs (Mbp) and black horizontal bars are marker positions

A genome-wide association analysis using single SNP markers with GAPIT’s FARMCPU method discovered 20 significant marker-trait associations (MTAs) in 9 chromosomes (Table 2**,** Fig. 3). The number of MTAs discovered per chromosome ranged from 1 to 3 with three chromosomes (2, 6, and 20) having three MTAs each. The largest MTA, i.e. MTA with highest proportion of phenotypic variance (R^2^) explained, was observed for a* (*R*^2^ = 11.2% for marker *S20_145384739*). Five MTAs had R^2^ ≥ 10% and the average R^2^ among all MTAs was 6.7% (median = 5.3%). Ten of the 20 MTAs contributed positive allelic effect towards the traits with largest allelic effect observed for b* (0.9, marker *S13_128050590*) and smallest allelic effect was observed for a* (− 1.8, marker *S02_183514050*). The mean minor allele frequency (MAF) of all significant SNP markers was 0.27. Four of the 20 significant SNP markers were common between two or more traits:

1. *S02_183514050* among L*, a*, and b*,
2. *S06_485505428* between L* and b*,
3. *S14_228811555* between L* and V, and
4. *S20_145384739* between a* and V.

**Table 2:** Single nucleotide polymorphism (SNP) markers in significant association with the CIELAB color traits L*, a*, b*, and V (visual score) for kernel color in the UMN_C4 intermediate wheatgrass population. The column ‘MAF’ lists minor allele frequency of the marker, ‘R^2^’ is the proportion of phenotypic variance explained by the significant SNP marker, and ‘Hb’ lists the haplotype block where the SNP marker was binned

Trait	SNP marker	Chr	Pos (Mbp)	Alleles	MAF	LOD	R^**2**^	Allelic effect	Hb
L*	S02_183514050	2	183.51	G/A	0.23	19.00	8.84	0.35	Chr02-Hb.145
L*	S06_485505428	6	485.51	C/G	0.50	6.80	4.26	−0.84	Chr06-Hb.193
L*	S14_228811555	14	228.81	T/C	0.49	18.46	10.50	−0.27	Chr14-Hb.64
L*	S16_133923040	16	133.92	A/G	0.21	6.66	4.70	−0.68	NA
L*	S20_435904483	20	435.90	G/A	0.15	6.16	4.35	0.69	NA
a*	S02_183514050	2	183.51	G/A	0.23	20.02	9.47	−1.84	Chr02-Hb.145
a*	S07_494007600	7	494.01	T/C	0.39	7.07	4.98	0.16	Chr07-Hb.208
a*	S13_272249905	13	272.25	T/C	0.44	8.33	5.84	−0.17	NA
a*	S20_145384739	20	145.38	G/A	0.20	19.57	11.19	−1.31	Chr20-Hb.107
b*	S02_183514050	2	183.51	G/A	0.23	16.03	10.94	0.43	Chr02-Hb.145
b*	S04_76206640	4	76.21	A/T	0.31	8.36	5.87	0.65	Chr04-Hb.82
b*	S04_105922911	4	105.92	T/C	0.18	5.85	4.14	0.83	NA
b*	S06_485505428	6	485.51	C/G	0.50	6.02	4.26	−0.76	Chr06-Hb.193
b*	S13_128050590	13	128.05	C/T	0.12	7.61	5.35	0.93	Chr13-Hb.106
V	S06_286239143	6	286.24	G/A	0.18	6.34	4.48	0.20	Chr06-Hb.116
V	S07_353011546	7	353.01	G/A	0.14	7.25	5.11	−0.17	Chr07-Hb.114
V	S10_457351151	10	457.35	A/C	0.07	6.02	4.26	−0.21	NA
V	S14_228811555	14	228.81	T/C	0.49	15.37	10.52	0.44	Chr14-Hb.64
V	S16_386540604	16	386.54	C/T	0.20	7.51	5.29	0.19	Chr16-Hb.184
V	S20_145384739	20	145.38	G/A	0.20	14.57	10.00	−0.76	Chr20-Hb.107

Fifteen of the 20 significant SNP markers were also binned into 10 haplotype blocks in 11 chromosomes (Table 2, column ‘Hb’**).** Of the 10 haplotype blocks, three were in significant association with the kernel color traits.

Genome-wide association analysis using multi-allelic haplotype blocks resulted in discovery of 23 significant haplotype-trait associations (HTAs) in 10 chromosomes (Table 3**,** Fig. 3). Chromosome 06 had the largest number of HTAs (5) followed by chromosome 17 (3). The largest HTA, i.e. HTA with the largest R^2^ value = 15% was *Chr14-Hb.64* and was located in chromosome 14. This block included the marker *S14_228811555* which was common between L* and V in the single marker analysis (Table 2). Nine HTAs had R^2^ ≥ 10% and the average R^2^ among all MTAs was 8.3% (median = 7.4). The correlation between R^2^ and the allelic effect of haplotype blocks was 0.51 and found to be significantly different (*P* = 0.003) from zero at *α* = 0.05. Six of the 23 significant HTAs were common between two or more traits:

1. *Chr02-Hb.145* between L* and a*,
2. *Chr06-Hb.193* among L*, b*, and V,
3. *Chr14-Hb.64* between L* and a*,
4. *Chr16-Hb.2* between L* and a*,
5. *Chr17-Hb.74* between L* and a*, and
6. *Chr21-Hb.125* between L* and a*.

**Table 3:** Haplotype blocks significantly associated with the CIELAB color traits L*, a*, b*, and V (visual score) for kernel color in the UMN_C4 intermediate wheatgrass population. The column ‘LOD’ is a -log10*P-value of marker-trait association, ‘R^2^’ is the proportion of phenotypic variance explained by the significant SNP marker, ‘SigSNP’ shows if a SNP marker binned in the significant haplotype block was also significant in the single marker analysis

Trait	Hb	Chr	SNPs in block	LOD	R^**2**^	Allelic effect	SigSNP
L*	Chr02-Hb.145	Chr02	2	12.93	12.08	−12.75	S02_183514050
L*	Chr06-Hb.193	Chr06	4	5.90	6.48	3.81	S06_485505428
L*	Chr06-Hb.212	Chr06	2	5.90	6.48	−8.57	NA
L*	Chr07-Hb.132	Chr07	7	6.11	11.98	−14.71	NA
L*	Chr13-Hb.231	Chr13	2	9.40	7.63	−8.34	NA
L*	Chr14-Hb.64	Chr14	4	11.41	14.89	−11.84	S14_228811555
L*	Chr16-Hb.2	Chr16	3	9.21	7.08	−6.72	NA
L*	Chr17-Hb.74	Chr17	4	11.83	13.34	−15.35	NA
L*	Chr21-Hb.125	Chr21	2	12.98	10.52	−14.38	NA
a*	Chr02-Hb.145	Chr02	2	6.10	6.28	2.29	S02_183514050
a*	Chr14-Hb.64	Chr14	4	6.64	10.17	3.32	S14_228811555
a*	Chr16-Hb.2	Chr16	3	11.56	8.21	−7.68	NA
a*	Chr17-Hb.74	Chr17	4	6.01	7.95	3.51	NA
a*	Chr21-Hb.125	Chr21	2	5.15	4.28	3.01	NA
b*	Chr06-Hb.193	Chr06	4	6.61	6.08	−3.98	S06_485505428
b*	Chr20-Hb.191	Chr20	2	5.23	5.53	−4.12	NA
V	Chr04-Hb.134	Chr04	2	12.72	12.35	−0.42	NA
V	Chr04-Hb.154	Chr04	2	10.83	10.19	−0.97	NA
V	Chr06-Hb.142	Chr06	2	5.61	6.08	−1.32	NA
V	Chr06-Hb.193	Chr06	4	5.25	10.17	2.88	S06_485505428
V	Chr07-Hb.60	Chr07	2	5.40	4.63	−2.05	NA
V	Chr17-Hb.30	Chr17	2	5.43	5.91	−1.00	NA
V	Chr20-Hb.114	Chr20	2	5.58	6.05	0.04	NA

Of the 24 significant HTAs, seven contained significant SNPs based on the single marker analysis (Table 3, column ‘SigSNP).

### Genomic prediction of kernel color

**Fig. 4:**
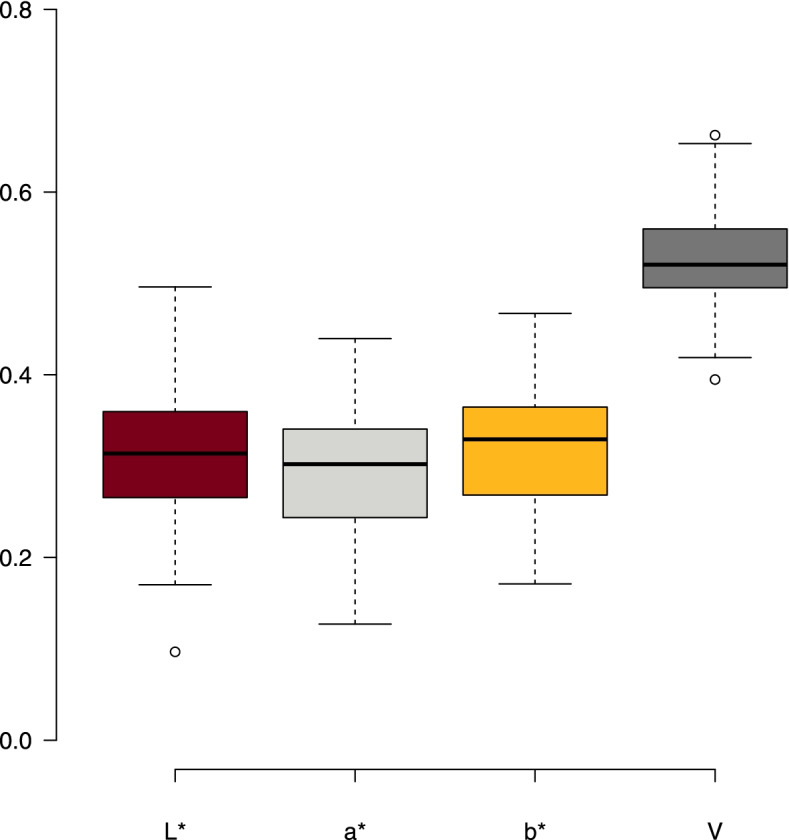
Predictive ability values (mean) for kernel color in IWG with the CIELAB kernel color traits L*, a*, b*, and visual score (V)

Genomic prediction of the kernel color traits in the UMN_C4 population carried out using four-fold cross validation showed that the visual score trait (V) had the overall best prediction ability, average *r*^*2*^ = 0.53. (Fig. 4). Mean predictive abilities for the CIELAB traits L*, a*, and b* ranged from 0.29–0.33.

### Identification and phylogeny of candidate genes

**Fig. 5:**
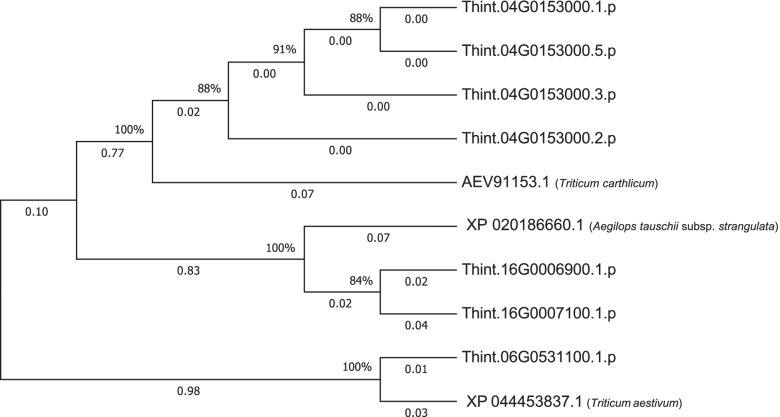
A Neighbor-Joining tree depicting the evolutionary relationships among protein coding sequences for MYB transcription factors in intermediate wheatgrass and other cereal species. Evolutionary distances displayed on the branches (i.e. branch lengths) are in the units of number of amino acid substitutions per site. The % values next to the branches indicate the proportion of replicate trees in which the associated taxa clustered together in the bootstrap test

A total of 260 protein coding sequences ≥100 bp were detected within 0.46 Mbp of all significant SNP markers and haplotype blocks in the *T. intermedium* v2.1 annotated genome. Of 260, 209 sequences had significant alignments (i.e. % identity ≥80% and E-value ≤1E-10) with protein sequences from other species following a BLAST search (Additional File 1). Further filtering of the alignments for functions associated with involvement in anthocyanin biosynthesis pathway resulted in discovery of seven unique *T. intermedium* protein coding sequences in three chromosomes (Table 4). Four of the seven IWG genes were discovered within 207 kilobase pairs (kbp) of the significant SNP marker *S04_76206640*; two were discovered within 39 kbp of the significant haplotype locus *Chr16-Hb.2*; and one (*Thint.16G0006900.1.p*) was located within 0.5 kbp of the significant SNP marker *S06_485505428*. An additional BLAST-search using the genes controlling grain color in wheat, *R-A1*, *R-B1*, and *R-D1*, found the genes *Thint.16G0006900.1.p* and *Thint.16G0007100.1.p*, both located within *Chr16-Hb.2*, to have weak resemblance to the wheat genes with % identity of 50–55% (Additional File 1). A phylogenetic analysis that used genes involved in MYB transcription factors revealed that the IWG genes: 1) from same chromosome clustered together, and 2) grouped closer to genes from other species with similar functions (Fig. 5).

**Table 4:** A summary of BLAST-search results showing significant alignments between intermediate wheatgrass candidate gene sequences and known genes in other species. In case of multiple alignments of an IWG gene to other genes in the NCBI database, the best alignment is shown per gene

IWG candidate gene	GWAS locus	Distance (kbp) from GWAS locus	Subject			% identity	E-value
			NCBI Gene ID	Species	Description		
*Thint.04G0153000.1.p*	*S04_76206640*	*207.21*	*AEV91153.1*	*Triticum carthlicum*	R2R3-MYB protein	92.7	0.0
*Thint.04G0153000.2.p*	*S04_76206640*	*207.21*	*AEV91153.1*	*Triticum carthlicum*	R2R3-MYB protein	92.5	0.0
*Thint.04G0153000.3.p*	*S04_76206640*	*207.21*	*AEV91153.1*	*Triticum carthlicum*	R2R3-MYB protein	92.6	0.0
*Thint.04G0153000.5.p*	*S04_76206640*	*207.21*	*AEV91153.1*	*Triticum carthlicum*	R2R3-MYB protein	88.3	0.0
*Thint.06G0531100.1.p*	*S06_485505428*	*0.51*	*XP_044453837.1*	*Triticum aestivum*	anthocyanin 3′-O-beta-glucosyltransferase-like	95.9	0.0
*Thint.16G0006900.1.p*	*Chr16-Hb.2*	*38.77*	*XP_020186660.1*	*Aegilops tauschii subsp. strangulata*	transcription factor MYB36	87.5	0.0
*Thint.16G0007100.1.p*	*Chr16-Hb.2*	*38.77*	*XP_020186660.1*	*Aegilops tauschii subsp. strangulata*	transcription factor MYB36	90.6	0.0

## Discussion

We quantified and genetically characterized kernel color in the fourth recurrent selection cycle (UMN_C4) population of intermediate wheatgrass at the University of Minnesota using the CIELAB method and visual rating. Visual scores (V) had higher broad-sense heritability estimate (H = 0.78) compared to L*, a*, and b* (H ranged from 0.41–0.53). As higher heritability value suggests that trait variation is largely influenced by genetic components, selection made using V will likely respond in population mean shifting towards the desired direction. Additionally, V was easier and faster to rate than the CIELAB components. As genomic predictions were also higher for V, it might be practical for a breeding program to use a visual method of phenotyping to select IWG genets for a specific kernel color or a range of colors. It should be noted however that higher genomic prediction estimates are often observed for traits with higher heritabilities, including in IWG [8, 27].

### Association mapping and genomic selection

Our GWAS discovered 20 single markers (SNPs) and 23 haplotype blocks associated with kernel color traits in IWG with few loci shared among the traits as well as between the two methods (Tables 2 and 3). The identified quantitative trait loci (QTL) explained small to medium proportions of observed trait distribution as the variance explained (R^2^) ranged from 4 to 11% for SNPs and 4–15% for haplotype blocks. Compared to other members of the Poaceae family, these values are similar to those reported in rice [12], barley [28], and durum wheat [29]. Since we identified genetic loci influencing kernel color traits in IWG for the first time, we were unable to compare our findings with other similar studies. However, we did compare the MTA and HTA positions with previously reported QTL in IWG to investigate if any loci were pleiotropic. We did not find any significant marker discovered in this study within the LD block (0.46 Mbp [3];) of previously reported markers in IWG. Additionally, as the QTL were of small to medium effects, several cycles of breeding and selection are likely necessary to increase the frequency of alleles conferring deep purple color to the grain, and fix them in the breeding population. The time needed to reach such milestone could be shortened by conducting recurrent selection simultaneously with marker-assisted selection and genomic selection [26].

Genomic selection is being increasingly used in crop breeding programs because of its potential in improving genetic gain per unit time while reducing cost [30]. Intermediate wheatgrass breeding programs in the US have adopted genomic selection based breeding and observed an overall positive trends in trait improvement [25, 27]. In this study we evaluated the possibility of using genomic selection to select for kernel color in an IWG population. The prediction models we implemented for this task showed that visual scores were predicted well in all environments whereas the L*, a*, and b* estimates varied by environment (Fig. 4). Using newer models, possibly ones that take into consideration genotype by environment (GxE) interaction, could further increase these predictions.

### Candidate genes and phylogeny

A search for candidate genes led to the discovery of four predicted protein sequences in IWG that had significant alignment to genes involved in regulation of the flavonoid synthesis pathway in other species (Table 4). Flavonoids are secondary metabolites in plants and contribute to various functions related to development and defense including fruit and flower colors and aroma, stress response, and disease resistance [31]. We discovered seven IWG genes located within a relatively short distance (< 210 kbp) that could potentially be involved in biosynthesis of MYB (myeloblastosis) transcription factor and anthocyanin 3′-O-beta-glucosyltransferase (3’GT). The MYB transcription factors are known to regulate flavonoid biosynthesis and are key determinants of pigmentation in seed/kernel, flower, and fruit [31, 32]. Similarly, anthocyanin 3’GT is involved in synthesis of blue anthocyanin in flower and grain including cereals [33, 34]. In particular, the IWG genes *Thint.16G0006900.1.p* and *Thint.16G0007100.1.p*, both positioned within the haplotype block *Chr16-Hb.2*, were found to resemble the genes *R-A1*, *R-B1*, and *R-D1* that control grain color in wheat (Additional File 1) [35]. It may therefore be possible that the significant haplotype locus *Chr16-Hb.2* harbors genes involved in anthocyanin biosynthesis pathway. Additional research is needed to definitively elucidate the function of these genes and the extent of their relationship with grain color expression in IWG.

As multiple IWG genes aligned to genes encoding for MYB transcription factors from other plant species, we explored the phylogenetic relationship among the MYB ortholog genes. A Neighbor-Joining tree revealed that the IWG genes from same chromosomes grouped together. We also observed that the IWG genes clustered based on functional similarity, i.e. they grouped with non-IWG genes than with other IWG genes from different chromosomes. In regards to evolutionary movement, it could be stated that the IWG genes presented in Fig. 5 are more divergent from other IWG genes based on their functional differences, despite all genes putatively characterized as being involved in pigment production pathways. In other words, there appears to be a strong evidence in favor of a speciation-like event among the IWG genes based on their functional differences.

### Implications in food applications and breeding

Fruits, vegetable, and edible grain with purple pigmentation are rich in anthocyanins that have a wide range of health benefits [36]. In grains, anthocyanin-rich maize has long been used by different cultures for their beneficial effects in human diets and also for ornamental purpose [37]. Purple wheat has been reported to have higher antioxidant and anti-inflammatory activity and could be used as a superior source of anthocyanin as well as a natural food colorant [36, 38]. As a novel grain crop with proven advantages in sustainable and regenerative agriculture, blue or purple IWG could also be a component in human dietary requirements. Already known to be high in protein content, carotenoids, and antioxidant content [39], IWG grain and flour have a combination of desirable features needed in making food products with a broad commercial reach. For example, pigmented bran and flour of mainstream annual crops such as wheat, barley, rice, and maize have been used to produce food products with blue/purple/black coloration [40–42]. Given the unique combination of flavor, nutritional profile, and functional characteristics of IWG grain [43], the prospect of developing colored IWG varieties for niche food applications and markets is highly appealing.

The University of Minnesota IWG breeding program currently does not select for a specific kernel color while making selection decisions and advancing generations. A separate breeding scheme could be implemented to select and maintain IWG germplasm with desired kernel color in case of a strong interest expressed by the food industry and consumers. One approach towards creation of such germplasm with improved frequency of causative alleles that contribute towards preferred kernel pigmentations could be the implementation of genomic selection as discussed earlier. Given the identification of potential candidate genes, as well as other significant loci influencing kernel color in IWG, a marker-assisted selection could also assist in identification of parental genotypes harboring beneficial alleles followed by crossing to accumulate these beneficial alleles.

## Conclusions

Our study genetically characterized kernel color traits in intermediate wheatgrass, which is the first study of this nature for this novel perennial grain crop. A broad range of phenotypic distribution was observed with medium to high heritability estimates. We identified 20 single SNP markers and 24 multi-allelic haplotype-based markers associated with kernel color traits measured visually and characterized by L*, a*, and b* values; several of these significant markers were shared among the traits. Genomic prediction of these traits suggested that a genomic selection based breeding approach to identify candidates for desired kernel color may be possible. In particular, as a perennial crop with a long cropping cycle, intermediate wheatgrass can benefit from application of genomic selection in selecting genotypes prior to their field-evaluation. Selection of candidates in this manner can potentially help in development of a separate pool of genotypes with desired kernel color with niche food applications.

## Methods

### Plant material

A population of 637 IWG genets was used in this study. A genet is defined as a genetically unique organism and refers to individual plants in an outcrossing species such as IWG [8]. The population, part of the fourth breeding selection cycle (referred to as UMN_C4 hereafter), is owned by the University of Minnesota and has been described in a previous report [3]. Briefly, UMN_C4 was obtained from open-pollination of 73 cycle 3 (UMN_C3) genets that were selected based on their superior genomic estimated breeding values (GEBVs) for larger seed size, better threshability, reduced seed shattering, higher grain yield, and reduced plant height. From each of the 73 mother plants, nine random seeds were germinated, cloned into two replicates, and transplanted in the field during June–September 2018 at two MN locations: Crookston and St. Paul. The transplants were distanced approximately 0.91 m (3 ft) apart and were surrounded on all sides with border IWG plants. The population was first harvested in August 2019 and then for a second time in August 2020 at both sites. The location and year combinations are abbreviated as follows in the text: Crk19: Crookston 2019, Crk20: Crookston 2020, StP19: St. Paul 2019, StP20: St. Paul 2020.

### SNP genotyping and haplotype construction

The population was genotyped following a reduced-representation sequencing method using the enzymes *PstI* and *MspI* [44] and sequenced on Illumina’s Novaseq 600. Sequenced reads were filtered for minimum read quality Q > 30 and aligned to the IWG reference genome v2.1 [45] using the ‘mem’ command in Burrows-Wheeler Aligner 0.7.5a [46]. Allele-calling was done using default parameters in SAMtools 1.6 and BCFtools 1.6 [47]. SNPs were filtered to keep those with minimum read depth per SNP site ≥5, minor allele frequency (MAF) of ≥3% and missing data ≤20%. This resulted in 25,909 genome-wide SNPs that were imputed using default parameters in Tassel 5.2.71 [48] using the LD-kNNi method [49].

The process of haplotype discovery has also been previously described [3]. In short, HAPLOVIEW 4.2 [50] was implemented to construct haplotype blocks for each chromosome and blocks were constructed using a confidence interval algorithm [51]. Haplotype blocks were converted to multi-allelic markers by assuming that allelic combinations in each block are independent alleles and blocks were numbered in ascending order (1,2,3 … n) if a block was not previously observed.

### Phenotyping and data analysis

To characterize kernel color in UMN_C4, mature spikes were harvested per plant and dried at 32 °C for 72 h. A Wintersteiger LD 350 (Wintersteiger Inc., Salt Lake City, USA) was used to thresh the spikes. Approximately 25 de-hulled seeds from each genet were first given a visual rating of 1–5 where 1 was assigned to the lightest color and 5 to the darkest color (Fig. 1). The same grains were then photographed using a Canon EOS Rebel T7 under ambient light conditions indoors. Kernel color in each seed was measured using the CIELAB method, which is recommended by the International Commission on Illumination (Commission Internationale de l’eclairage, CIE) for its perceptually uniform color space [52]. In this method, three numerical values are estimated as L* (lightness/darkness), a* (redness/greenness), and b* (yellowness/blueness) which was done using ImageJ 1.53e [53]; values were averaged among all seed per genet. The process of image analysis and measurement of L*, a*, and b* values in ImageJ is also shown in Fig. 1 and the macro used as well as obtained phenotypic data are available in Additional File 1.

For each trait, the best linear unbiased estimates (BLUEs) were calculated across all trials, i.e. combinations of two locations in 2 years, using the following model:

$${\mathrm{Y}}_{\mathrm{i}\mathrm{j}\mathrm{k}}=\mu +{\mathrm{G}}_{\mathrm{i}}+{\mathrm{L}}_{\mathrm{j}}+{\mathrm{Y}}_{\mathrm{k}}+{\left(\mathrm{GL}\right)}_{\mathrm{i}\mathrm{j}}+{\left(\mathrm{GY}\right)}_{\mathrm{i}\mathrm{k}}+{\left(\mathrm{LY}\right)}_{\mathrm{j}\mathrm{k}}+{\mathrm{E}}_{\mathrm{i}\mathrm{j}\mathrm{k}}$$

where, Y_ij_ is the trait observation for genet i at location j in year k, μ is the mean, G_i_ is the main effect for genet I, Lj is the main effect at location j, Y_k_ is the main effect in year k, (GL) ij is the genotype by location interaction effect for genet i at location j, (GY) ij is the genotype by year effect for genet i in year k, (LY) ij is the location by year effect at location j in year k, and E_ijk_ is the error effect. The BLUEs were calculated using the R package ‘lme4’ and were used in all analyses, i.e. phenotypic correlations among the traits, association mapping, and genomic prediction.

Broad-sense heritability (H) on a genet-mean basis were estimated using:

$$\mathrm{H}={\sigma_{\mathrm{G}}}^2/\left({\sigma_{\mathrm{G}}}^2+{\sigma_{\mathrm{G}\mathrm{L}}}^2/\mathrm{L}+{\sigma_{\mathrm{G}\mathrm{Y}}}^2/\mathrm{Y}+{\sigma_{\mathrm{E}}}^2/\mathrm{L}\mathrm{Y}\right)$$

where, σ_G_^2^ is the genetic variance, σ_GL_^2^ is the genotype by location variance, σ_GY_^2^ is the genotype by year variance, σ_E_^2^ is the residual variance, L is number of locations, and Y is number of years.

### Association analysis

Genome-wide association study (GWAS) with single SNP marker data was done with the ‘FarmCPU’ method in R 4.0.2 [54, 55]. Association analysis with multi-allelic haplotype data was carried out in Tassel 3 with a mixed linear model with optimum compression level and variance component estimated using the P3D method (population parameters previously determined) [48]. To control for population structure during the GWAS with both SNP markers and haplotype data, a kinship matrix was used in the GWAS models. Additional need to control for population structure was evaluated using up to 10 PCs using the ‘Model.selection’ option. Results showed that the optimal number of PCs to use in the model was zero (Additional File 1) and therefore no PCs were used in the final GWAS models.

Significant quantitative trait loci (QTL) were declared at default Bonferroni thresholds at *α* = 0.05, i.e.

A. At α/no. of total observations = 0.05/25909 = *P* value of 1.93E-06 or LOD equivalent of 5.71 for single SNP markers
B. At α/no. of total observations = 0.05/5379 = *P* value of 9.30E-06 or LOD equivalent of 5.03 for multi-allelic haplotype blocks/markers

The percentage of phenotypic variation explained by significant markers (R^2^) in both GWAS analyses were calculated using the method of Sen and Churchill [56] as implemented in the ‘qtl’ R package [57].

### Candidate gene search and phylogenetic analysis

To search for candidate genes in regions surrounding the significant SNP markers and haplotype blocks, putative protein coding sequences within 0.46 megabase pairs (Mbp) of the significant loci were obtained from the *T. intermedium* v2.1 annotation [45]. The distance of 0.46 Mbp was used because the genome-wide average linkage disequilibrium in UMN_C4 was found to be 0.23 Mbp [3]. A protein BLAST-search was carried out on NCBI’s website [58] using the *T. intermedium* protein coding sequences with % identity > 80% and E-value ≤1E-10; only the best 10 alignments per *T. intermedium* protein coding sequence were retained for further analysis. These alignments were then filtered to scan for candidate genes known to control color pigmentation in cereal grains, e.g. MYB (myeloblastosis) transcription factors and anthocyanin-related enzymes. In this study, we focus on MYB transcription factors because of their role in the anthocyanin biosynthesis pathway that render blue or purple grain control in small grains [59, 60]. An additional BLAST-search was done using the protein sequences of three wheat genes *R-A1*, *R-B1*, and *R-D1* that are known to control grain color in this crop [35].

Evolutionary analyses and tree construction among the candidate gene sequences were done in MEGA7 with 10,000 bootstrap steps [61]. Evolutionary distances among the protein coding sequences were estimated using the Poisson correction method [62] assuming substitution rates among sites were uniform. After removal of positions with gaps and missing information during the analysis, a total of 353 positions were used in the final dataset.

### Evaluation of genomic prediction

The effectiveness of genomic prediction models in predicting IWG kernel color was evaluated using the R package ‘rrBLUP’ [63]. Correlations (*r*) between predicted trait value and BLUEs were calculated using a four-fold cross-validation method where 75% of the UMN_C4 panel was used as the training set and the remaining 25% was used as the validation set. The training and validation sets were sampled randomly without replacement and correlations were averaged from 100 replications.

## Supplementary Information

**Additional file 1.** Scripts and additional tables generated during the course of the study. There are four sheets in this file. Name and description of each sheet is provided below: ImageJ macro: Macro script used in the ImageJ program to obtain L*, a*, and b* values from seed images. Phenotypic data: L*, a*, b* values obtained from ImageJ and visual rating scores pre-data adjustment. Trait variance components: Genetic and non-genetic variance components generated using a mixed model equation. Narrow sense heritabilities: Narrow sense trait heritability estimates for the kernel color traits. PC model selection BIC values: BIC values calculated by GAPIT when using up to 10 PC axes as cofactors in the GWAS model to evaluate model fit. NCBI BLAST results: Protein BLAST results when intermediate wheatgrass candidate gene sequences were searched against the NCBI protein database. BLAST with wheat R genes: BLAST results when intermediate wheatgrass candidate gene sequences were searched against the three genes in wheat known to be involved in controlling kernel color in wheat.

## Data Availability

Sequence data of UMN_C4 can be accessed through NCBI’s SRA Bio-project PRJNA722274 (https://www.ncbi.nlm.nih.gov/bioproject?term=PRJNA722274). All other data generated and analyzed during this study are included in this published article and its additional information file.

## Abbreviations

BLUE: Best linear unbiased estimates
HTA: Haplotype-trait association
IWG: Intermediate wheatgrass
LD: Linkage disequilibrium
MTA: Marker-trait association
QTL: Quantitative trait locus
SNP: Single nucleotide polymorphism
